# EMOTIONALLY MEANINGFUL GOALS IN LATE ADULTHOOD

**DOI:** 10.1093/geroni/igae098.0372

**Published:** 2024-12-31

**Authors:** Nicole Fung, Helene H Fung

**Affiliations:** The Chinese University of Hong Kong, Hong Kong, China (People’s Republic); The Chinese University of Hong Kong, Hong Kong, China (People’s Republic)

## Abstract

Our perception of time greatly affects what kinds of goals we value and pursue. The socioemotional selectivity theory (SST; Carstensen, 1992; Carstensen et al., 1999) posits that people rearrange their goals in response to a limited future time perspective as they become older. Rather than investing in a limited and uncertain future, older adults will value and prioritize present-oriented emotionally meaningful goals over future-oriented goals. The SST literature thrived in the past three decades and emotionally meaningful goals were operationalized in various ways under different contexts. To better understand the changes in motivation when future time perspective becomes limited, we reviewed the empirical articles that formulated hypotheses according to the SST framework published since 1992. We identified different types of emotionally meaningful goals in the literature. The most prevalent type of emotionally meaningful goal in the literature is the pro-hedonic goal (preference towards positivity over negativity). Other types of emotionally meaningful goals include the pro-closeness goal (preference towards emotional closeness in social relationships), the prosocial and generativity goal (increased motivation to contribute to others), the social goal (increased importance in the social domain), the emotional goal (increased importance in the emotional domain), the self-maintenance goal (increased motivation for the intrinsic and self-concordant goal) and the meaningfulness goal (increased importance in meaningfulness). The empirical evidence of each goal will be reviewed and their relations to SST and a limited future time perspective will be discussed.

